# Self-assembly of cholesterol end-capped polymer micelles for controlled drug delivery

**DOI:** 10.1186/s12951-020-0575-y

**Published:** 2020-01-15

**Authors:** Ming Gao, Yifeng Yang, Andreas Bergfel, Lanli Huang, Li Zheng, Tim Melander Bowden

**Affiliations:** 1 Guangxi Engineering Center in Biomedical Materials for Tissue and Organ Regeneration, The First Affiliated Hospital of Guangxi Medical University, Nanning, 530021 China; 2 Guangxi Collaborative Innovation Center for Biomedicine, The First Affiliated Hospital of Guangxi Medical University, Nanning, 530021 China; 3 Department of Chemistry-Ångström Laboratory, Uppsala University, Box 538, 75121 Uppsala, Sweden; 4 Pharmaceutical College, Guangxi Medical University, Nanning, 530021 China

**Keywords:** Atom transfer radical polymerization, Supermolecular self-assembly, Amphiphilic polymer micelles, Critical micelle concentration, Controlled drug delivery system

## Abstract

**Background:**

During the past few decades, drug delivery system (DDS) has attracted many interests because it could enhance the therapeutic effects of drugs and reduce their side effects. The advent of nanotechnology has promoted the development of nanosized DDSs, which could promote drug cellular uptake as well as prolong the half-life in blood circulation. Novel polymer micelles formed by self-assembly of amphiphilic polymers in aqueous solution have emerged as meaningful nanosystems for controlled drug release due to the reversible destabilization of hydrophobic domains under different conditions.

**Results:**

The amphiphilic polymers presented here were composed of cholesterol groups end capped and poly (poly (ethylene glycol) methyl ether methacrylate) (poly (OEGMA)) as tailed segments by the synthesis of cholesterol-based initiator, followed by atom transfer radical polymerization (ATRP) with OEGMA monomer. FT-IR and NMR confirmed the successfully synthesis of products including initiator and polymers as well as the Mw of the polymers were from 33,233 to 89,088 g/mol and their corresponding PDI were from 1.25 to 1.55 by GPC. The average diameter of assembled polymer micelles was in hundreds nanometers demonstrated by DLS, AFM and SEM. The behavior of the amphiphilic polymers as micelles was investigated using pyrene probing to explore their critical micelle concentration (CMC) ranging from 2.53 × 10^−4^ to 4.33 × 10^−4^ mg/ml, decided by the balance between cholesterol and poly (OEGMA). Besides, the CMC of amphiphilic polymers, the quercetin (QC) feeding ratio and polarity of solvents determined the QC loading ratio maximized reaching 29.2% certified by UV spectrum, together with the corresponding size and stability changes by DLS and Zeta potential, and thermodynamic changes by TGA and DSC. More significantly, cholesterol end-capped polymer micelles were used as nanosized systems for controlled drug release, not only alleviated the cytotoxicity of QC from 8.6 to 49.9% live cells and also achieved the QC release in control under different conditions, such as the presence of cyclodextrin (CD) and change of pH in aqueous solution.

**Conclusions:**

The results observed in this study offered a strong foundation for the design of favorable polymer micelles as nanosized systems for controlled drug release, and the molecular weight adjustable amphiphilic polymer micelles held potential for use as controlled drug release system in practical application.

## Background

Many drugs have unacceptable side effects due to unwanted interactions with non-targeted parts of the body [1, 2]. These side effects hamper the ability to design optimal medical regimes for treatment of diseases and call for the development of drug delivery system (DDS) strategies. DDS, referring to enhance therapeutic effects of drug molecules and reduce their related side effects, attracts many interests during the past few decades [3, 4]. An ideal DDS would control the localization, presentation and release of active drugs in the target tissue or cellular compartment within a predefined concentration during a specified period of time.

The advent of nanotechnology has promoted the design of nanoparticles, [5, 6] nanocapsules, [7, 8] polymer micelles [9, 10] and liposome [11, 12] as nanosized DDSs. Specifically, polymer micelles derived from the self-assembly of amphiphilic polymers has sparked an interest as DDSs since they can be prepared in nanometer size, in order to promote cellular uptake and with the potential benefit of an increased half-life in blood circulation [13, 14]. However, the formation of polymer micelles is mostly built by the structural design of the polymer where hydrophobic and hydrophilic segments are combined in so called amphiphilic polymers [15]. Their hydrophobicity can be reached by the introduction of hydrophobic groups such as fluorination, [16, 17] cholesterol, [18, 19] ferrocene, [20, 21] pyrene, [22, 23] polyesters [24, 25] and these groups will form domains which can encapsulate lipophilic therapeutic drugs. Meanwhile, their hydrophilic segments could be selected from natural polymers such as polysaccharides, [26] polypeptides, [27, 28] or synthetic polymers including poly (ethylene glycol) (PEG) and PEG-ylated polymers [15, 29]. Herein, an important parameter for polymer micelle formation is the critical micelle concentration (CMC), which mainly depends on the balance between hydrophilic and hydrophobic segments. Besides, the types of hydrophobic group, molecular weight and distribution of hydrophilic parts in the amphiphilic polymer also influence the CMC as well as the stability of polymeric micelle and the drug loading ratio [30]. For instance, cholesterol is a hydrophobic molecule and natural component of the cell membrane, widely used in liposomes with good biocompatibility [18, 19, 31]. Finally, strategies for controlled release from DDS aim for creating reversible conditions by destabilizing the micelles at intracellular conditions including lowering of pH, [32, 33] temperature, [7] glutathione, [34, 35] specific enzymes, [5, 36] and external factors: light, [37] magnetic field, [38, 39] electric field [20, 21] and other molecules [40, 41]. In this respect, amphiphilic polymer micelles could be used to optimize DDSs by chemically modulating the core-shell structure of micelles in order to match selected drugs and the physiological conditions [9, 10].

**Fig. 1:**
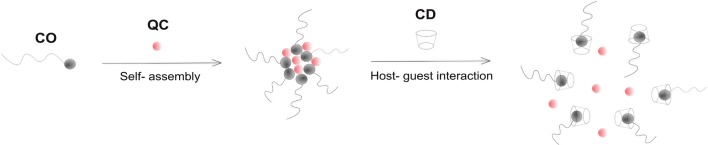
Schematic image of self-assembly of cholesterol-poly (OEGMA) amphiphilic polymer (CO)-quercetin (QC) micelles and their host–guest interaction mediated destabilization with β-cyclodextrin and subsequent drug release

Motivated by these findings, we present the preparation of amphiphilic cholesterol end-capped poly (poly (ethylene glycol) methyl ether methacrylate)s, abbreviated as CO polymers, use them to form polymer micelles and evaluate their effects as controlled DDS by the presence of β-cyclodextrin or change of pH. The polymers are comprised of a hydrophilic outer shell of poly (poly (ethylene glycol) methyl ether methacrylate), abbreviated as poly (OEGMA), and a hydrophobic inner core of cholesterol and can form nanoscale micelles in aqueous medium by self-aggregations. Atom transfer radical polymerization (ATRP) is used to synthesize the amphiphilic polymers with controlled polymer molecular weight and narrow molecular weight distribution, [42] which could significantly affect the critical micelle concentration (CMC) and drug loading ratio. Here quercetin (QC) is selected as a model drug due to its anti-tumor activity, reported inhibition of allergic and inflammatory responses of the immune system, and preventing growth of bacteria and fungi plus being a vasoprotective and an antithrombotic agent [24]. The polymer micelles are expected to encapsulate the hydrophobic drug and perform a controlled release affected by either pH or the induced host–guest interaction between cholesterol and β-cyclodextrin. Figure 1 illustrates the schematic procedures of self-assembly of CO-QC polymer micelles and their host–guest interaction with β-cyclodextrin to reach controlled drug release.

## Experimental section

### Materials and equipment

Cholesterol (≥ 99%), α-bromoisobutyryl bromide (98%), triethylamine (≥ 99%), 2,2-bipyridyl (bpy, ≥ 99%), magnesium sulfate (≥ 99.5%), silica gel (high-purity grade, pore size 60 Å, 200–425 mesh particle size), copper (II) chloride (CuCl_2_, ≥ 99%), copper (I) chloride (CuCl_,_ ≥ 99.99%), anisole (99.7%), oligo (ethylene glycol) methyl ether methacrylate (OEGMA, average M_n_ = 500 g/mol), aluminum oxide (Al_2_O_3_ basis, ≥ 98%), pyrene (99%), quercetin (QC, ≥ 95%), β-cyclodextrin (≥ 97%) were obtained from Sigma-Aldrich. Solvents (diethyl ether, methylene chloride (DCM), methanol, tetrahydrofuran (THF) and acetone) were purchased from VWR and in analytical grade. Chemicals were used without further treatment with the exceptions for OEGMA that was purified by passing through basic Al_2_O_3_ to remove the inhibitor and the recrystallization of QC from acetone.

The synthesized cholesterol initiator and polymers were characterized by Fourier transform infrared spectra (FTIR, PerkinElmer spectrum One FT-IR spectrometer), ^1^H NMR spectra (400 MHz, Jeol JNM-ECP Series FT NMR) using deuterated chloroform (CDCl_3_) as solvent and gel permeation chromatography (GPC, Agilent Technologies, 1260 infinity). Atomic force microscopy (AFM) and scanning electron microscopy (SEM) was recorded on Nanosurf Mobile S, TS150 and Leo 1550 SEM instrument Zeiss, Germany respectively. Particle size and distribution was investigated using a zeta sizer nano instrument from Malvern Instruments, UK. Thermal properties were studied on lyophilized samples by thermogravimetric analysis on TGA Q500, and differential scanning calorimetry on DSC Q1000 (TA instruments).

### Synthesis of CO polymers

Cholesterol end-capped polymers were synthesized using a two-step procedure. In step one a cholesterol initiator (Chol-Br) was synthesized. Briefly, cholesterol (1.0 g, 2.6 mmol) was dissolved in 150 ml of diethyl ether followed by the addition of triethylamine (0.47 ml, 3.4 mmol). The mixture was placed on an ice bath and α-bromoisobutyryl bromide (0.72 g, 1.34 ml, 3.1 mmol) was added dropwise. After two hours a white ammonium salt precipitate was filtered off by vacuum filtration. Additional solvent (CH_2_Cl_2_) was added and the organic phase was washed for three times with water, dried with MgSO_4_ and filtered. The organic phase was further passed through a plug of silica, evaporated and additionally dried in vacuum to afford the Chol-Br as a white solid. In the second step, the amphiphilic cholesterol end-capped poly (OEGMA) (CO) was prepared by ATRP as follows: cholesterol-Br (0.021 g) was dissolved in anisole (5 ml) after combining with CuCl_2_ (0.27 mg) and bpy (0.013 g). Subsequently a certain amount of OEGMA monomer (8 g) was added to the mixture. The final mixture was vacuumed to remove oxygen by repeated freezing, pump and thawing steps. The reaction mixture was heated at 60 °C for 24 h with monitoring the reaction progress by NMR. After reaction, the mixture firstly was quenched with acetone, before filtered through a silica gel column followed by the basic Al_2_O_3_ column to remove excess copper elements as previously done [43, 44]. The final product was precipitated in diethyl ether before vacuum drying. A series of polymers varying in degree of polymerization were produced using the same protocol and were defined as CO50, CO100, and CO200 by the feed molar ratio between cholesterol-Br and OEGMA.

### Preparation of quercetin (QC) loaded polymer micelles

Polymer micelles with or without QC were prepared as follows. The polymer and a predetermined amount of QC were separately dissolved in different solvent (acetone, DCM, methanol or THF). Then the obtained mixture was added dropwise to PBS buffer under stirring to form the polymer complexes overnight. After evaporation of solvents, the final solutions were dialyzed (Spectra/Por® 6 Dialysis Membrane, MWCO: 3.5 KD) against deionized water for 24 h to remove the free QC to obtain the QC-loaded polymer micelle solution.

### Measurement of critical micelle concentration (CMC) of polymer micelles

The CMC measurements of polymer micelles were implemented by fluorescent microscopy (Luminescence Spectrometer, LS45, PerkinElmer Instrument). Pyrene was used as a fluorescence probe to analyze the polymer micelles in PBS buffer (pH = 7.4). Samples for fluorescent microscopy were prepared as previously described [45, 46]. Cholesterol end-capped polymers and pyrene were dissolved in the methanol before the drop-wise addition of PBS buffer. The mixture was stirring overnight to reach the total evaporation of solvents. The concentration of polymers ranged from 1 × 10^−6^ to 0.1 mg/ml while the pyrene concentration was chosen to 4.5 × 10^−5^ mg/ml in the final solution. The slit widths for both excitation and emission sides were maintained at 2.5 nm and excitation and emission wavelength of 339 nm and 374 nm were respectively applied.

### Determination of QC loading ratio

The polymer-QC micelle solutions were mixing with THF with the volume ratio of 1: 1. The final solutions were investigated by UV–Vis spectroscopy (Lambda 35 UV/Vis spectrometer, PerkinElmer Instrument) at the wavelength of 380 nm. The QC standard curve was obtained by measuring the QC solutions (THF: PBS buffer with volume ratio of 1: 1) of various concentrations. Finally, the QC loading ratio was calculated by the obtained absorbance based on the QC standard curve.

### Cell cytotoxicity of polymer and polymer micelles

Mouse myoblast cells (C2C12) were cultured in Dulbecco's modified eagle medium (DMEM, Life technologies) supplemented with 10% fetal bovine serum (FBS, Life technologies) and 0.1% penicillin-streptomycin to near 80% confluence. Cells were maintained at 37 °C with 5% CO_2_ at 90% humidity for 24 h and then seeded in 96-well plates with the density of 8000 cells per ml. Cells were replaced with fresh medium and then respectively added the corresponding sample solutions (polymer micelles, polymer-QC micelles or QC) to reach total 100 μl medium in each of wells. After incubated for another 24 h, the cells were washed with PBS buffer and 100 μl 1% alamar blue solution (Life technologies) was added to each well. The plate was incubated at 37 °C, 5% CO_2_ for 2 h. The absorbance was measured at 570 nm in a microplate reader (Tecan infinite M200). The final results were compared with control wells to determine the relative cell viability.

### In vitro investigation of QC release

Polymer-QC micelle samples (0.5 ml) were saved in dialysis tube (Slide-A-Lyzer MINI Dialysis Devices, 3.5 K MWCO, Fisher) and suspended in a cut-off of 15 ml centrifuge tube (4.5 ml release medium). The release study was performed at room temperature in an incubator shaker. At selected time intervals, solutions (0.5 ml) outside of dialysis tube were removed and combined with the same volume of methanol as released samples, and then replaced with fresh release medium. The concentrations of QC in release samples were analyzed by high performance liquid chromatography (HPLC, WATERS e2695) with a 2489 UV/Vis detector. QC samples with different concentration were applied to prepare the standard curve. HPLC was running at gradient mode by the mobile phase of methanol-phosphoric acid buffer (0.7% in milli-Q water) with volume ratio from 0: 100 to 80: 20. The flow rate was 1 ml/min and elution time was 9 min for each sample with C18 column (X Bridge 3.5 m, 4.6 × 50 mm Column, WATERS). The absorbance was chosen at 360 nm wavelength with the elution time of 0.75 min.

## Results and discussion

### Synthesis of cholesterol-Br and amphiphilic polymers

**Fig. 2:**
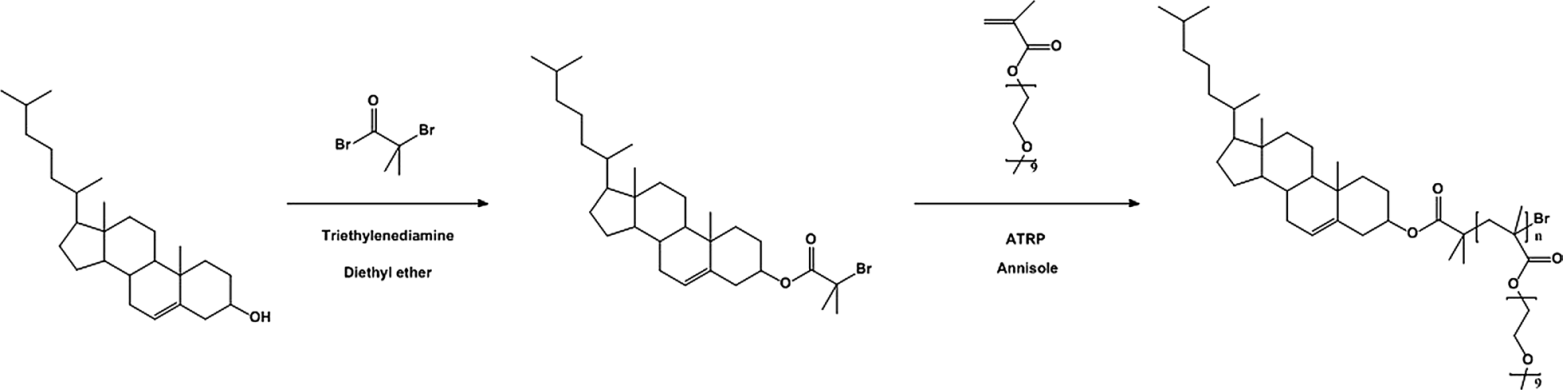
Schematic steps of preparing cholesterol initiator (cholesterol-Br) and cholesterol-poly (OEGMA) amphiphilic polymer (CO)

The amphiphilic cholesterol end-capped polymers were synthesized by atomic transfer radical polymerization (ATRP). Its synthetic procedures were schematically illustrated in Fig. 2. The cholesterol-Br was prepared by the reaction between cholesterol and α-bromoisobutyryl bromide to form the initiator for ATRP. Subsequently, the amphiphilic polymer (CO) was synthesized by ATRP using the bpy as the ligand and anisole as the solvent at 70 °C. From GPC data, the Mw and PDI for synthesized polymers were respectively: CO50 (Mw = 33,233 g/mol, PDI = 1.25), CO100 (Mw = 52,168 g/mol, PDI = 1.32), and CO200 (Mw = 89,088 g/mol, PDI = 1.55).

### FT-IR and ^1^H NMR of cholesterol-Br and amphiphilic polymers

**Fig. 3:**
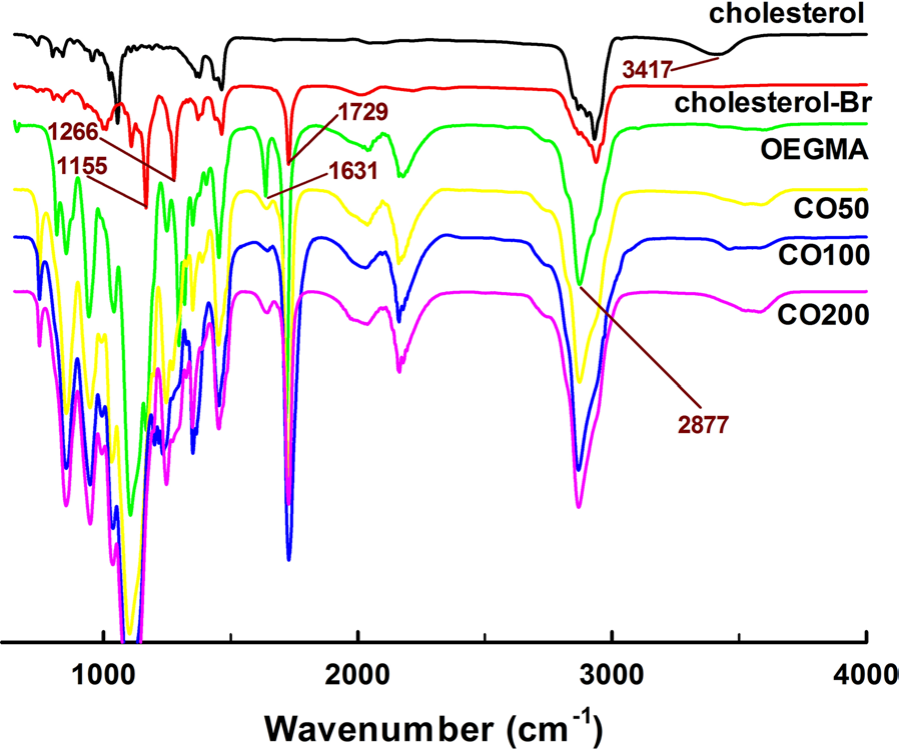
FT-IR of samples: cholesterol, cholesterol-Br, OEGMA, CO50, CO100 and CO200

The cholesterol initiator and polymers were characterized by FT-IR and NMR. Figure 3 illustrated the FT-IR results of the chemical samples. The peak at 3417 cm^−1^ was OH groups for cholesterol while it disappeared in cholesterol-Br. The appearance of peak at 1729 cm^−1^ (C=O) also gave the proof of successful formation of cholesterol-Br. Besides, the peaks at 1631 cm^−1^ and 2877 cm^−1^ respectively corresponded to C=C and –CH stretch vibration of OEGMA monomer. It finally confirmed the successful preparation of amphiphilic polymers (CO50, CO100 and CO200) due to the inclusion of the peaks at 1729, 1266 and 1155 cm^−1^ for cholesterol-Br and 2877 cm^−1^ for OEGMA.

**Fig. 4.**
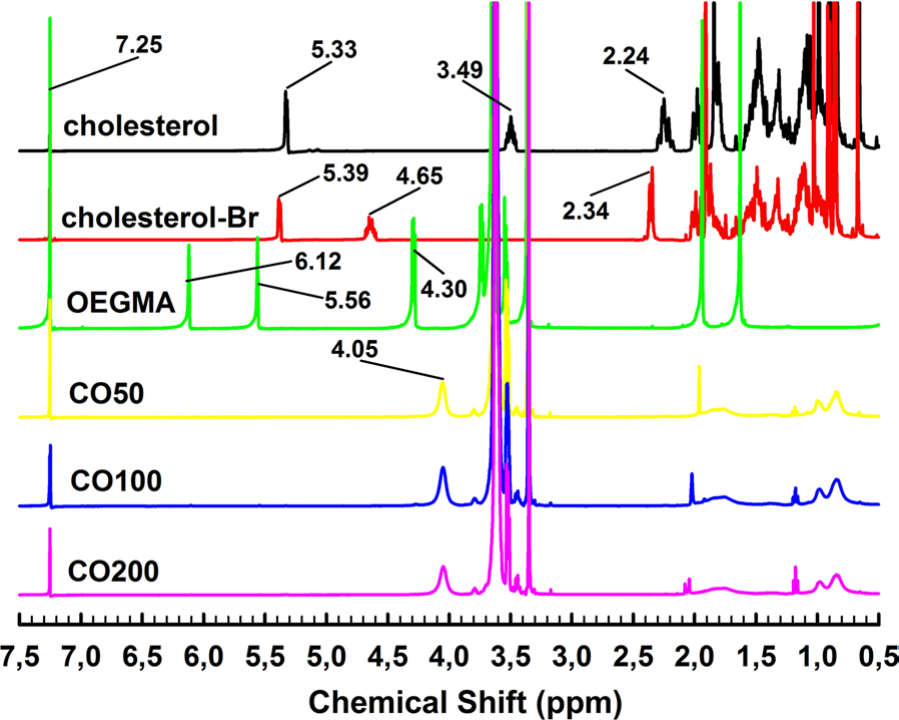
^1^H NMR of samples: cholesterol, cholesterol-Br, OEGMA, CO50, CO100 and CO200 with solvent of CDCl3

The corresponding ^1^H NMR results were shown in Fig. 4 with the solvent peak of CDCl_3_ at 7.25 ppm. The observed proton (C=C) was at 5.33 ppm for pristine cholesterol and a slight shift at 5.39 ppm for cholesterol-Br. And the chemical shift at 3.49 ppm was corresponding to the OH groups of cholesterol not observed after modification for cholesterol-Br. The specific peak observed at 4.65 ppm was the proton of isobutyryl groups for cholesterol-Br. In addition, the chemical shifts were respectively shown at 5.56 and 6.12 ppm (protons of C=C), and 4.30 ppm (protons of –CH_2_–) for OEGMA monomer. The final cholesterol end-capped polymer structure was confirmed using ^1^H NMR, characterized by protons of –CH_2_– at 4.05 ppm for OEGMA and protons ranging from 0.5 to 2 ppm for cholesterol. The disappearance of protons at 5.56 and 6.12 ppm provided the information of total polymerization of OEGMA monomers.

### Thermal properties of polymers and polymer-QC micelles

The thermal properties of initiator, amphiphilic polymers, and polymer-QC complexes were investigated by TGA and DSC. Additional file 1: Figure S2 illustrated the weight loss ratio of cholesterol, cholesterol-Br, OEGMA monomer and CO polymers from 26 °C to 600 °C under nitrogen gas atmosphere. The decomposition temperature of cholesterol-Br was 252.3 °C, a slight increase from unmodified cholesterol (250.8 °C). The OEGMA monomer had a broad decomposition temperature range from 150 °C to 400 °C while the corresponding decomposition temperature of synthesized CO polymers were respectively 283.1 °C for CO50, 291.3 °C for CO100 and 303.3 °C for CO200. The results showed that the decomposition temperature increased with the increase of polymer molecular weight. From the DSC results (Additional file 1: Figure S3), the apparent melting curve was observed at 39.2 °C for cholesterol while it shifted to 121.9 °C after modification to form cholesterol-Br. The increase of repeated units in CO polymers contributed to the close thermal ability between OEGMA monomer and CO polymers. Additional file 1: Figure S4 illustrated the TGA results of CO100-QC micelles. The melting curve was clearly observed at 68.9 °C for drug QC and its weight loss ratio was around 63% during the temperature range from 26 to 600 °C. Mostly, the weight loss ratios were 100%, 97%, 95% and 91.6% for CO100-QC respectively with the CO: QC weight ratio of 20: 1, 10: 1, 5: 1 and 2: 1 as well as the weight loss ratio of pure CO100 was nearly 100%. Increasing the drug loading ratio induced higher decomposition temperature and lower weight loss ratio due to the increased amount of QC in the CO-QC complexes. Furthermore, it was also apparently presented that the melting peaks became more obvious and closer to pure QC with the increase of QC amount from CQ: QC = 20: 1 to 2: 1 (Additional file 1: Figure S5).

### CMC of polymer micelles

**Fig. 5:**
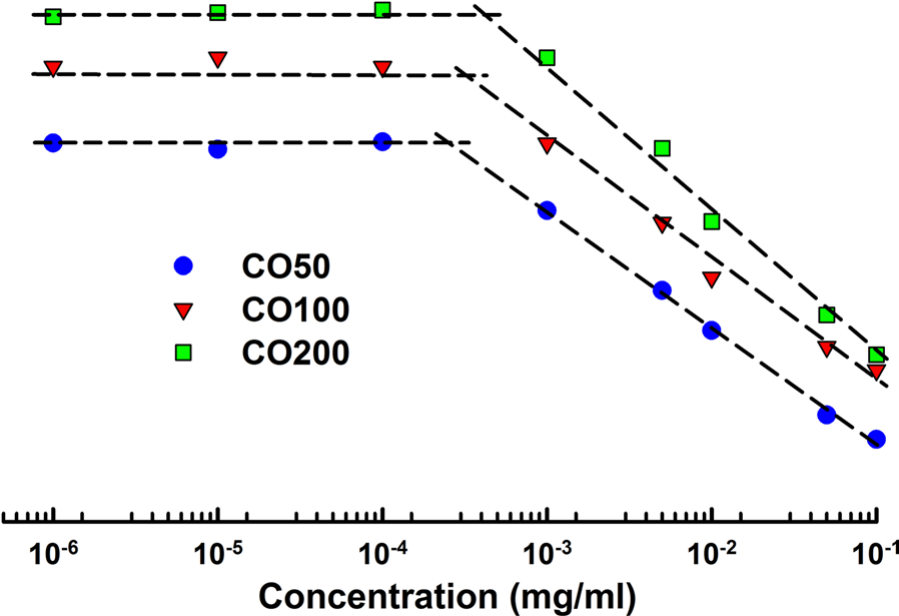
Plot of I388/I401 (from pyrene emission spectra by fluorescent microscopy) versus the concentration of CO50, CO100 and CO200 ranging from 1 × 10^−6^ to 0.1 mg/ml

CMC is one of the significant parameters for the preparation of polymer micelles as DDSs in biomedicines. Above CMC, the amphiphilic polymers were expected to form polymer micelles in water by self-assembly. Furthermore, the fluorescent intensity of Pyrene is a molecular sensitive to the polarity of hydrophobic microenvironment and would be utilized as the fluorescent probe. At last the CMC was obtained by linear regression of the plotted functions A_388nm_/A_401nm_ from fluorescent microscopy. The original intensity versus concentration of CO100-pyrene was listed in Additional file 1: Figure S1. The fluorescent intensity increased with the increase of CO concentration. The ratios between selected intensities at 388 nm and 401 nm were plotted as the function to determine the CMC of polymer micelles (Fig. 5). Therefore, the CMC of CO50, CO100 and CO200 were respectively 2.53 × 10^−4^, 3.28 × 10^−4^ and 4.33 × 10^−4^ mg/ml. The longer hydrophilic repeated units required higher concentration of amphiphilic polymer to form polymer micelles and further induced the higher CMC of polymers.

### Size and zeta potential of polymer and polymer-QC micelles

The concentration of polymer micelles was 0.01 mg/ml in the final solution of PBS buffer (pH = 7.4). The size and zeta potential of polymer micelles and polymer-QC micelles were initially analyzed by Zeta sizer at room temperature. Additional file 1: Figure S6 presented the size and zeta potential of different polymers-QC micelles with the weight ratio of 5: 1 prepared by dissolving them in THF after solvent evaporation in PBS buffer. The size and zeta potentials were 243.0 ± 15.1 nm and − 9.5 ± 0.3 for CO50 micelles while they became 184.9 ± 9.2 nm and − 13.0 ± 2.1 for CO100, and 155.9 ± 7.1 nm and − 12.2 ± 0.5 for CO200. It seemed that the higher molecular weight (Mw) of polymers always provided the smaller size and lower zeta potential of polymer micelles. After QC loading in CO50, the corresponding size and zeta potential changed to 377.3 ± 14.7 nm and − 17.1 ± 1.9. It was the same situation that the size of polymer micelles increased, and their corresponding zeta potential decreased after QC loading in polymer micelles for CO100 and CO200. Higher molecular weight means the longer hydrophilic chain of CO polymers, which could contribute not only the smaller size but also stronger stability of polymer micelles.

The weight ratio of CO100: QC was also expected to affect the size and zeta potential of polymer micelles. From Additional file 1: Figure S7, the size was 192.0 ± 24.5 nm, 263.8 ± 27.6 nm, 385.7 ± 55.6 nm and 534.4 ± 15.5 nm for polymer-QC complexes with CO100: QC weight ratio from 20: 1, 10: 1, 5: 1 and 2: 1. Their zeta potentials also gently decreased from − 18.3 ± 0.8 for 20: 1 to − 24.7 ± 1.4 for 2: 1. The solubility of polymers and QC in different solvents also had the effects on size and zeta potential of micelles. Additional file 1: Figure S8 illustrated the size and zeta potential of polymer micelles prepared by dissolving polymer and/or QC in different solvents after the evaporation of solvents in PBS buffer (pH = 7.4). The size and corresponding zeta potential respectively was 134.4 ± 15.5 nm and − 21.2 ± 2.1 for methanol, 184.9 ± 9.2 nm and − 13.0 ± 2.1 for THF, 225.8 ± 35.0 nm and − 11.90 ± 1.6 for acetone, and 235.1 ± 31.4 nm and − 3.6 ± 0.8 for DCM. The good solubility was helpful to provide homogeneous dispersion of polymers so that the size of polymer micelles became smaller and their stabilities also increased in the presence of increased absolute value of zeta potential. Correspondingly the poor solvents induced the irregular aggregation of polymers and destabilized the polymer micelles.

**Fig. 6:**
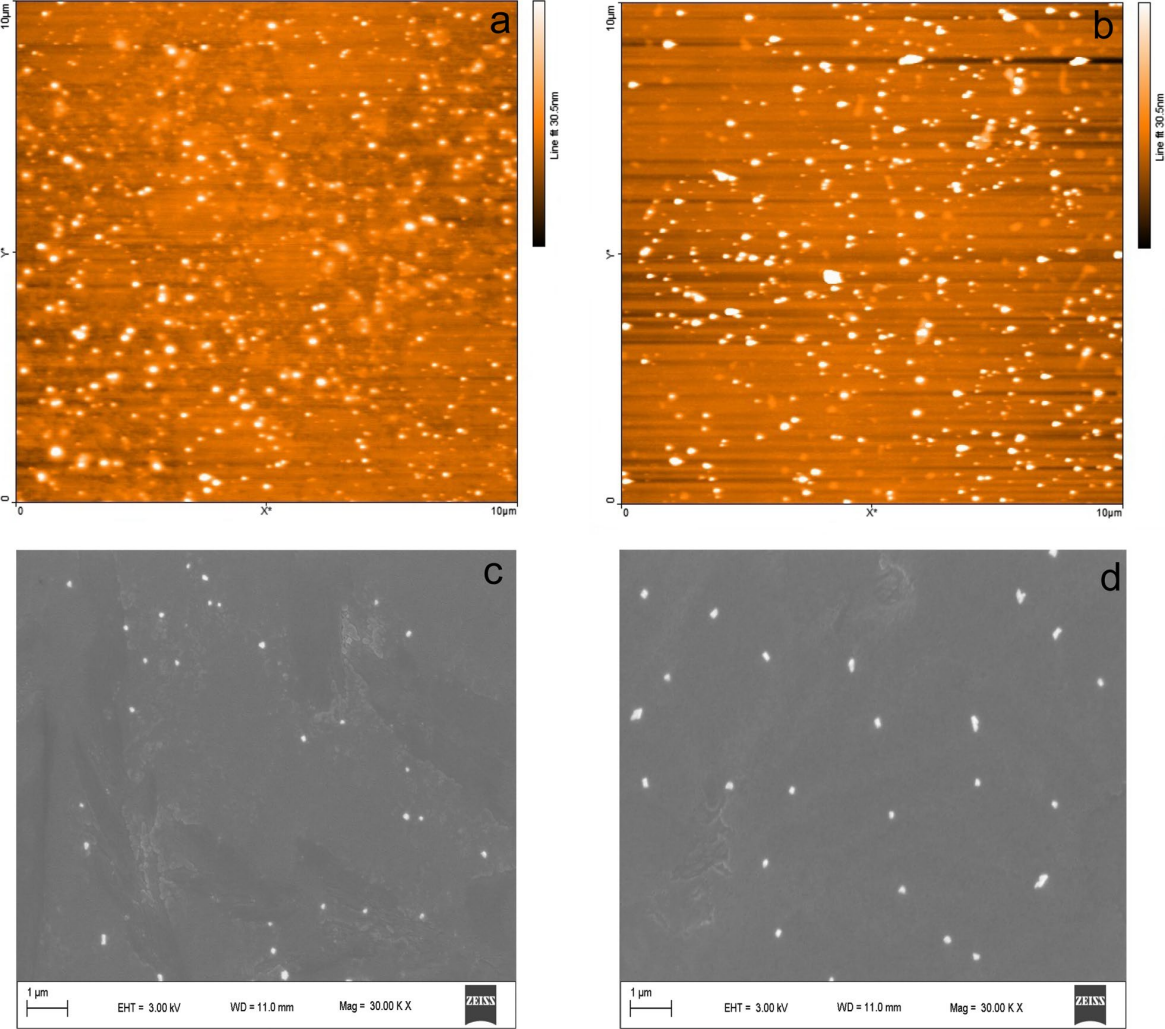
AFM and SEM images of CO100 (**a**, **c**) and CO100-QC (weight ratio of 5: 1, **b**, **d**) micelles prepared by dissolving the CO100 and/or QC in THF after solvent evaporation in DI water to reach 0.01 mg/ml CO100 in the final solution

In addition, the size of CO and CO-QC micelles was investigated by SEM and AFM. Figure 6 illustrated the AFM and SEM results of CO100 and CO100-QC (weight ratio of 5: 1) micelles in DI water. The size of pure CO100 micelles was below 200 nm in diameter (Fig. 6a, c) while the size obviously increased (200–300 nm) after QC loading with the weight ratio of 5: 1 (Fig. 6b, d), which was the same conditions for CO50 before and after QC loading with the same weight ratio (Additional file 1: Figures S9 and S10). The size of micelles became bigger (around 300 nm) by increasing QC weight to CO: QC = 2: 1 (Additional file 1: Figure S11).

### Drug loading ratio of polymer micelles

**Fig. 7:**
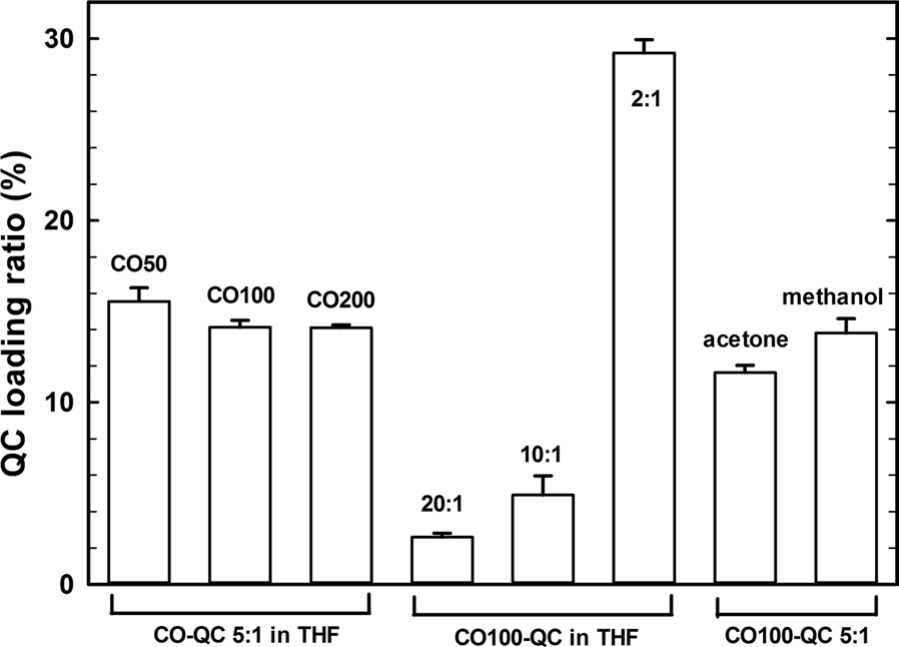
QC loading ratio of CO micelles prepared by different types of CO polymers (CO50, CO100 and CO200) respectively dissolved in different solvents (acetone, methanol and THF) with different CO: QC weight ratio of 20: 1, 10: 1, 5: 1 and 2: 1 after solvents evaporation to reach the CO concentration of 0.1 mg/ml in the final solution

The drug-loading ratio was obtained by calculating UV absorbance based on the QC standard curve. From Fig. 7, the drug loading ratio was 15.56 ± 0.75% for CO50, which slightly decreased to 14.13 ± 0.39% for CO100 and 14.10 ± 0.16% for CO200 with CO: QC weight ratio of 5: 1 and prepared by dissolving CO and QC in THF after solvents evaporation in PBS buffer (pH = 7.4). If increasing the CO: QC weight ratio from 20: 1, 10: 1 to 2: 1, their QC loading ratio respectively became 2.60 ± 0.12%, 4.91 ± 1.06% and 29.21 ± 0.73%. However, QC loading ratio was 11.64 ± 0.39% and 13.81 ± 0.80% by separately changing the solvent to acetone and methanol (weight ratio of 5: 1). Good solvents for CO and QC were also beneficial to increase the drug loading.

### Cell cytotoxicity of polymer-QC micelles

**Fig. 8:**
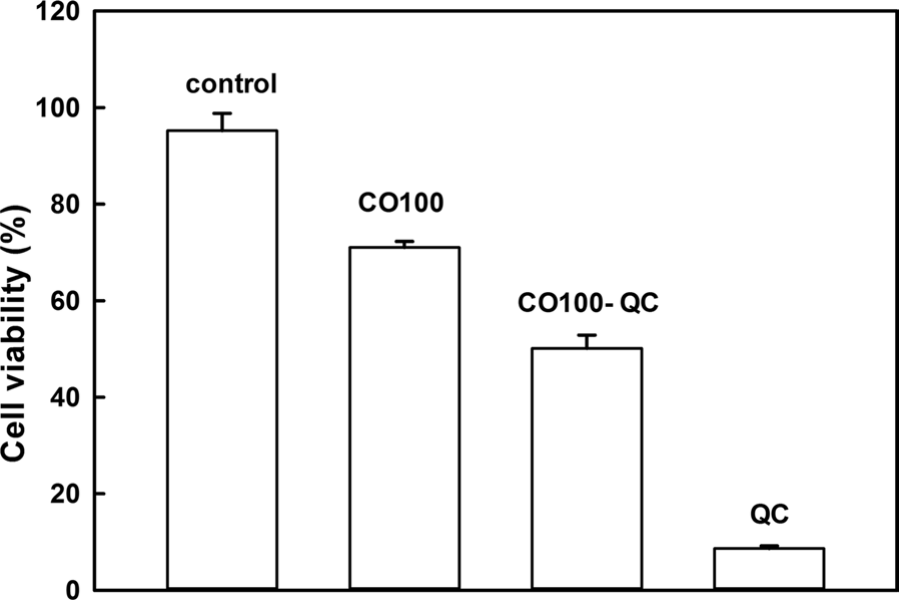
C2C12 cell viability cultured with 0.1 mg/ml CO100, 0.1 mg/ml CO100-QC micelles (CO100: QC = 5: 1), and 0.02 mg/ml QC in the final solution after incubation for 24 h

The aim of developing DDSs is to reduce the cytotoxicity of drugs and avoid the deletion of medicinal effect. The cell cytotoxicity of CO-QC micelles was investigated by mixing them with cultured cells for 24 h. For free QC medium, 91.4% of cells were dead after 24 h of incubation. The cell viability of CO100-QC micelles was 50.1% while the pure CO100 micelle still killed 29.9% of cells. Therefore, QC loaded polymer micelles could effectively decrease the toxicity of pure QC under the same condition. (Fig. 8).

### In vitro release of QC

**Fig. 9:**
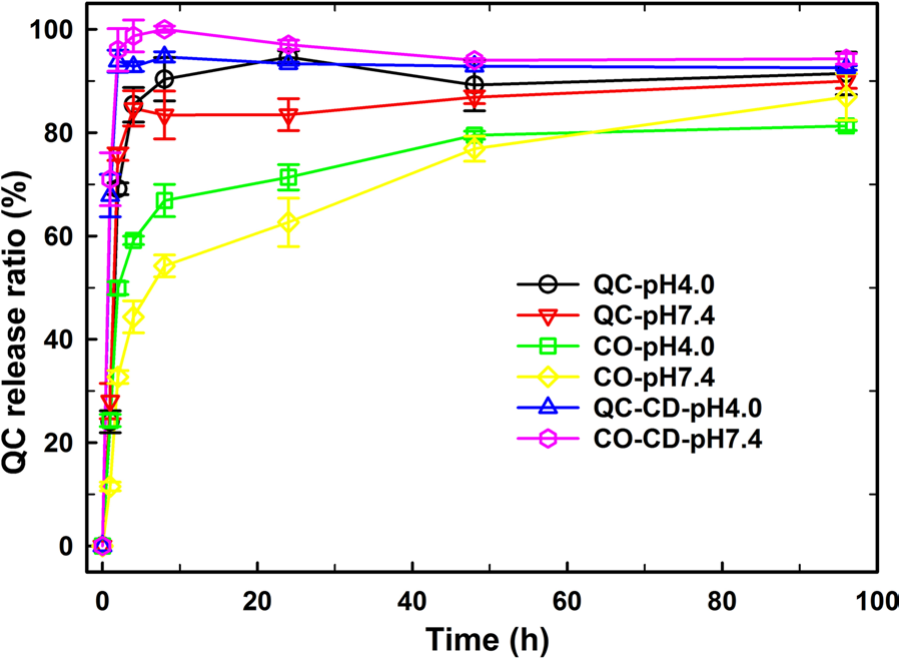
In vitro QC release ratio of different samples under different defined medium. They are: free QC (0.02 mg/ml) in PBS buffer of pH = 4 (QC-pH4.0) and pH = 7.4 (QC-pH7.4), CO100-QC (0.1 mg/ml CO100, CO: QC = 5: 1) micelles in PBS buffer of pH = 4.0 (CO-pH4.0) and pH = 7.4 (CO-pH7.4), and CO100-QC (5: 1) micelles in pH = 4.0 and pH = 7.4 PBS buffer with 0.1 mg/ml β-cyclodextrin (CO-QC pH4.0 and CO-QC pH7.4)

The release of QC from defined medium was monitored by UV detector of HPLC and the release ratio was calculated based on obtained UV absorbance of 0.02 mg/ml QC in the corresponding defined medium by HPLC. The total release time of free QC was within 24 h for pH = 4.0 PBS buffer (QC-pH4.0 in Fig. 9) while it changed to below 4 h for pH = 7.4 PBS buffer (QC-pH7.4). After forming CO100-QC polymer micelles (CO100: QC = 5: 1, 0.1 mg/ml CO100 in final solution), the release time simultaneously grew to above 48 h for both pH = 4.0 (CO-pH4.0) and pH = 7.4 (CO-pH7.4) PBS buffer. However, the addition of β-cyclodextrin (0.1 mg/ml in final solution) significantly induced the fast release of QC. Therefore, the release time of CO-QC samples became below 8 h for PBS buffer with β-cyclodextrin (QC-CD-pH4.0 and QC-CD-pH7.4). QC was more soluble in pH = 7.4 PBS buffer than pH = 4.0 PBS buffer so that QC was easily released in higher pH PBS buffer. The formation of CO-QC micelles resulted in the slow release of QC in PBS buffer. However, the quick QC release was achieved by host–guest interaction between cholesterol groups of CO and β-cyclodextrin which could destabilize the hydrophobic domain of cholesterol and QC.

## Conclusions

The cholesterol end-capped amphiphilic polymers were applied to form quercetin (QC) loaded micelles by self-assembly in aqueous solution after solvent evaporation to obtain controlled DDSs. TGA, DSC and UV spectroscopy were used to investigate the QC loading ratio determined by CMC of polymer micelles, weight ratio between amphiphilic polymer and QC as well as the solubility of polymers/QC in different solvents. It was found that the size of polymer-QC micelles was in hundreds nm scale by DLS, AFM and SEM, which could be easily uptake by cultured cells. It also demonstrated that in vitro release of polymer-QC micelles not only alleviated the cytotoxicity of QC, but was controlled under different conditions, e.g. pH and presence of cyclodextrins in the released medium. The results observed in this study offered a strong foundation for the design of favorable polymer micelles as systems for controlled drug release.

## Supplementary information

**Additional file 1: Figure S1.** Fluorescent intensity of CO100-pyrene micelles prepared by dissolving CO100 and pyrene in THF after solvent evaporation in PBS buffer (0.01 mg/ml CO100 in final solution, pH = 7.4). **Figure S2.** TGA results of cholesterol, cholesterol-Br, OEGMA monomer and amphiphilic polymers CO50, CO100 and CO200. The heating speed is 5 °C/min from room T to 600 °C under N_2_ atmosphere. **Figure S3.** DSC results of cholesterol, cholesterol-Br, OEGMA monomer and amphiphilic polymers CO50, CO100 and CO200. The heating flow is 10 °C/min from room T to 200 °C. **Figure S4.** TGA results of CO100, QC and CO100-QC complexes with different CO: QC weight ratio of 20: 1, 10: 1, 5: 1 and 2: 1. The heating speed is 5 °C/min from room T to 600 °C under N_2_ atmosphere. **Figure S5.** DSC results of CO100, QC and CO100-QC complexes with different CO: QC weight ratio of 20: 1, 10: 1, 5: 1 and 2: 1. The heating flow is 10 °C/min from room T to 200 °C. **Figure S6.** Size and zeta potential of different types of CO and CO-QC micelles with the CO: QC weight ratio of 5: 1 in PBS buffer (pH = 7.4). The concentration of CO polymers is 0.01 mg/ml in the final solution. **Figure S7.** Size and zeta potential of CO100 and CO100-QC micelles with different CO: QC weight ratio of 20: 1, 10: 1, 5: 1 and 2: 1 in PBS buffer (pH = 7.4). The concentration of CO polymers is 0.01 mg/ml in the final solution. **Figure S8.** Size and zeta potential of CO100 and CO100-QC complexes with CO: QC weight ratio of 5: 1 prepared by dissolving the CO100 and/or QC in different solvents (acetone, DCM, methanol and THF) after solvents evaporation in PBS buffer (pH = 7.4). The concentration of CO polymers is 0.01 mg/ml in the final solution. **Figure S9.** SEM image of CO50 polymer micelles prepared by dissolving CO50 in THF after solvent evaporation in DI water to reach 0.01 mg/ml CO50 in the final solution. **Figure S10.** SEM image of CO50-QC micelles (CO: QC = 5: 1) prepared by dissolving CO50 and QC in THF after solvent evaporation in DI water to reach 0.01 mg/ml CO50 in the final solution. **Figure S11.** SEM image of CO100-QC micelles (CO: QC = 2: 1) prepared by dissolving CO100 and QC in THF after solvent evaporation in DI water to reach 0.01 mg/ml CO100 in the final solution.

## Data Availability

All data generated or analyzed during this study are included in this published article.

## Abbreviations

ATRP: atom transfer radical polymerization
CMC: critical micelle concentration
QC: quercetin
DDS: drug delivery system
Mw: weight-average molecular weight
PDI: polymer disperse index
