# Supplementary material for: Self-assembly of cholesterol end-capped polymer micelles for controlled drug delivery
Source: J Nanobiotechnology. 2020 Jan 15;18:13. doi: 10.1186/s12951-020-0575-y (PMC6964014; doi:10.1186/s12951-020-0575-y)
Supplement: Supplementary file 1 — Additional file 1: Figure S1. Fluorescent intensity of CO100-pyrene micelles prepared by dissolving CO100 and pyrene in THF after solvent evaporation in PBS buffer (0.01 mg/ml CO100 in final solution, pH = 7.4). Figure S2. TGA results of cholesterol, cholesterol-Br, OEGMA monomer and amphiphilic polymers CO50, CO100 and CO200. The heating speed is 5 °C/min from room T to 600 °C under N2 atmosphere. Figure S3. DSC results of cholesterol, cholesterol-Br, OEGMA monomer and amphiphilic polymers CO50, CO100 and CO200. The heating flow is 10 °C/min from room T to 200 °C. Figure S4. TGA results of CO100, QC and CO100-QC complexes with different CO: QC weight ratio of 20: 1, 10: 1, 5: 1 and 2: 1. The heating speed is 5 °C/min from room T to 600 °C under N2 atmosphere. Figure S5. DSC results of CO100, QC and CO100-QC complexes with different CO: QC weight ratio of 20: 1, 10: 1, 5: 1 and 2: 1. The heating flow is 10 °C/min from room T to 200 °C. Figure S6. Size and zeta potential of different types of CO and CO-QC micelles with the CO: QC weight ratio of 5: 1 in PBS buffer (pH = 7.4). The concentration of CO polymers is 0.01 mg/ml in the final solution. Figure S7. Size and zeta potential of CO100 and CO100-QC micelles with different CO: QC weight ratio of 20: 1, 10: 1, 5: 1 and 2: 1 in PBS buffer (pH = 7.4). The concentration of CO polymers is 0.01 mg/ml in the final solution. Figure S8. Size and zeta potential of CO100 and CO100-QC complexes with CO: QC weight ratio of 5: 1 prepared by dissolving the CO100 and/or QC in different solvents (acetone, DCM, methanol and THF) after solvents evaporation in PBS buffer (pH = 7.4). The concentration of CO polymers is 0.01 mg/ml in the final solution. Figure S9. SEM image of CO50 polymer micelles prepared by dissolving CO50 in THF after solvent evaporation in DI water to reach 0.01 mg/ml CO50 in the final solution. Figure S10. SEM image of CO50-QC micelles (CO: QC = 5: 1) prepared by dissolving CO50 and QC in THF after solvent evaporat [file 12951_2020_575_MOESM1_ESM.docx]

**Additional Figures**


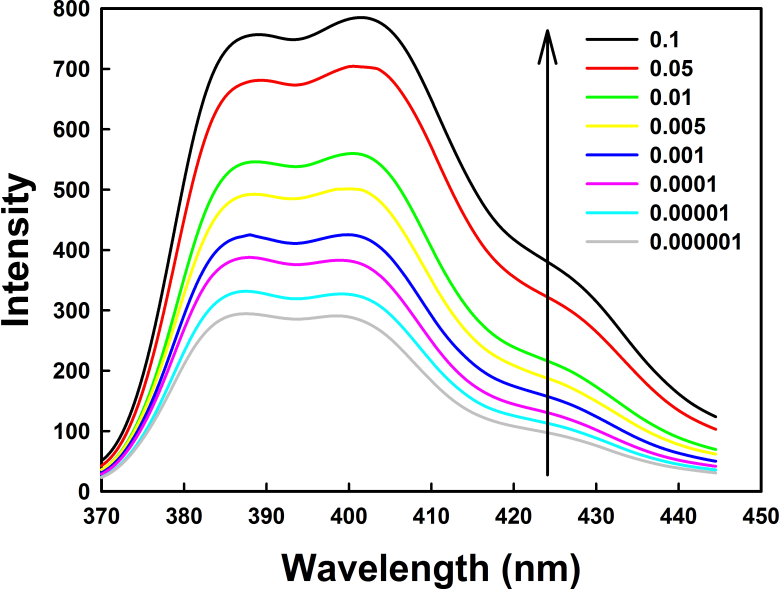


**Figure s1.** Fluorescent intensity of CO100- pyrene micelles prepared by dissolving CO100 and pyrene in THF after solvent evaporation in PBS buffer (0.01mg/ ml CO100 in final solution, pH= 7.4).


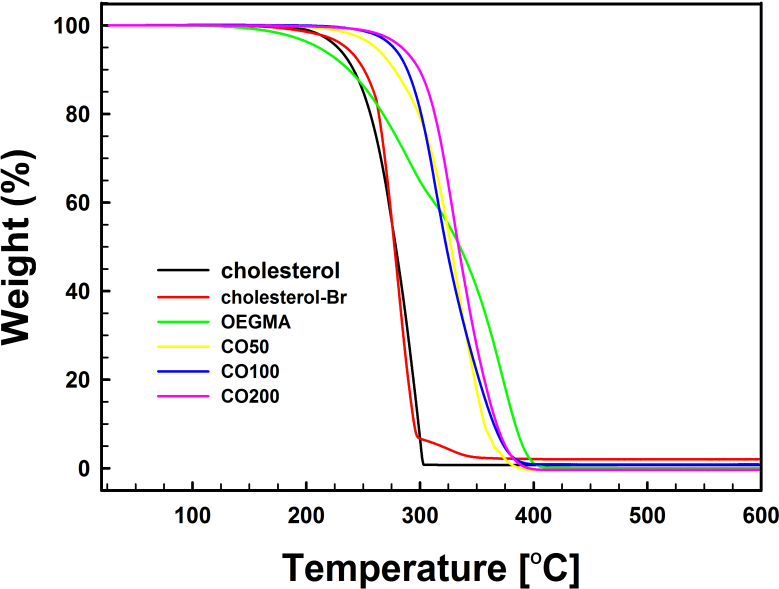


**Figure s2.** TGA results of cholesterol, cholesterol- Br, OEGMA monomer and amphiphilic polymers CO50, CO100 and CO200. The heating speed is 5 ℃/ min from room T to 600 ℃ under N_2_ atmosphere.


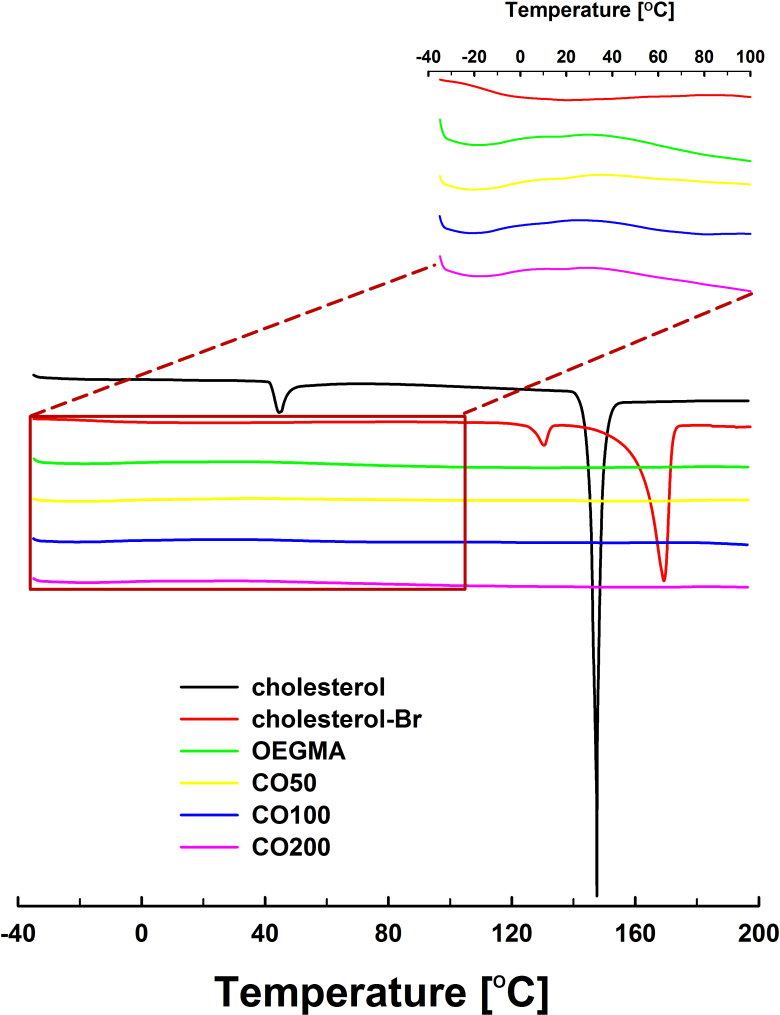


**Figure s3.** DSC results of cholesterol, cholesterol- Br, OEGMA monomer and amphiphilic polymers CO50, CO100 and CO200. The heating flow is 10 ℃/ min from room T to 200 ℃.


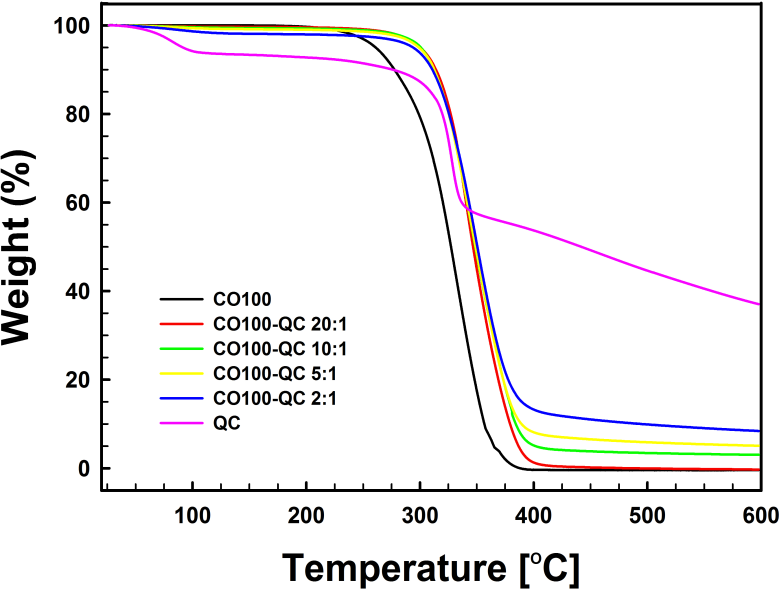


**Figure s4.** TGA results of CO100, QC and CO100- QC complexes with different CO: QC weight ratio of 20: 1, 10: 1, 5: 1 and 2: 1. The heating speed is 5 ℃/ min from room T to 600 ℃ under N_2_ atmosphere.


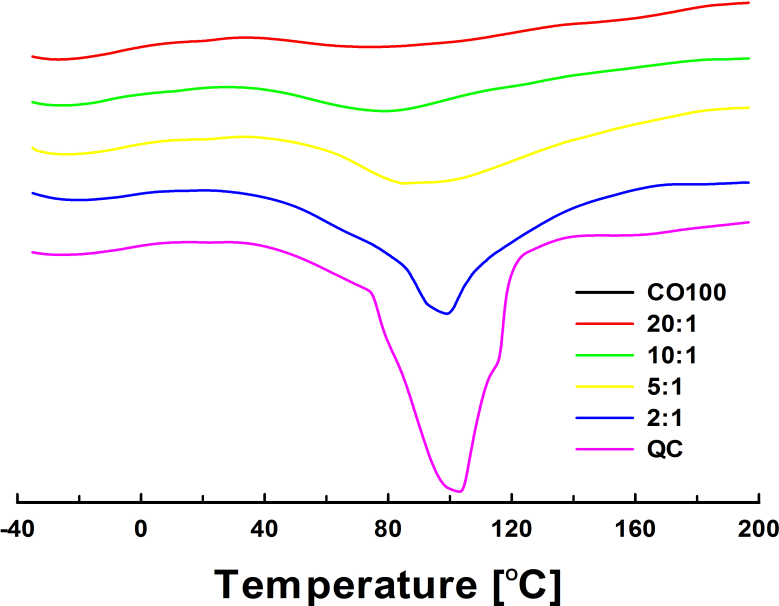


**Figure s5.** DSC results of CO100, QC and CO100- QC complexes with different CO: QC weight ratio of 20: 1, 10: 1, 5: 1 and 2: 1. The heating flow is 10 ℃/ min from room T to 200 ℃.


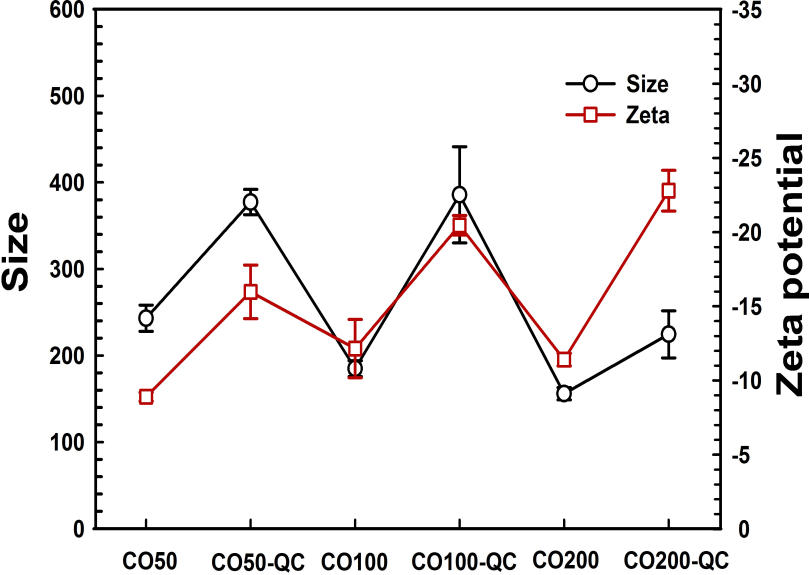


**Figure s6.** Size and zeta potential of different types of CO and CO- QC micelles with the CO: QC weight ratio of 5: 1 in PBS buffer (pH= 7.4). The concentration of CO polymers is 0.01 mg/ ml in the final solution.


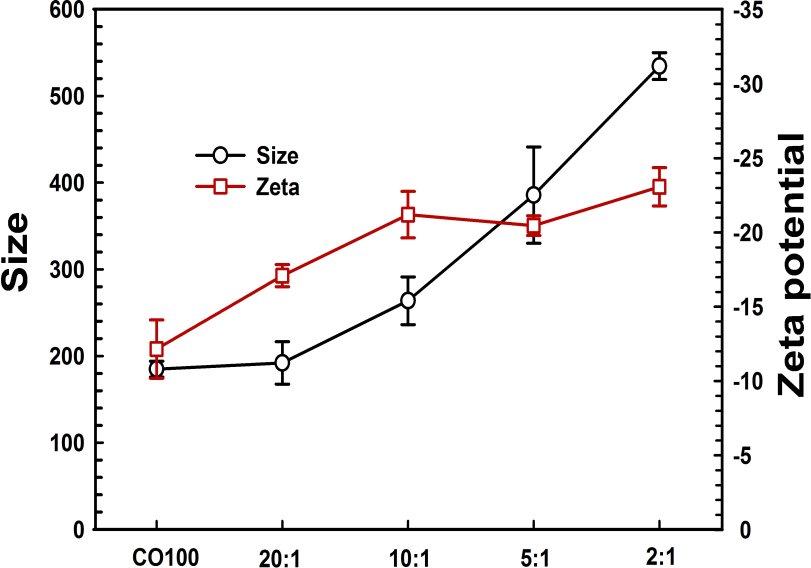


**Figure s7.** Size and zeta potential of CO100 and CO100- QC micelles with different CO: QC weight ratio of 20: 1, 10: 1, 5: 1 and 2: 1 in PBS buffer (pH= 7.4). The concentration of CO polymers is 0.01 mg/ ml in the final solution.


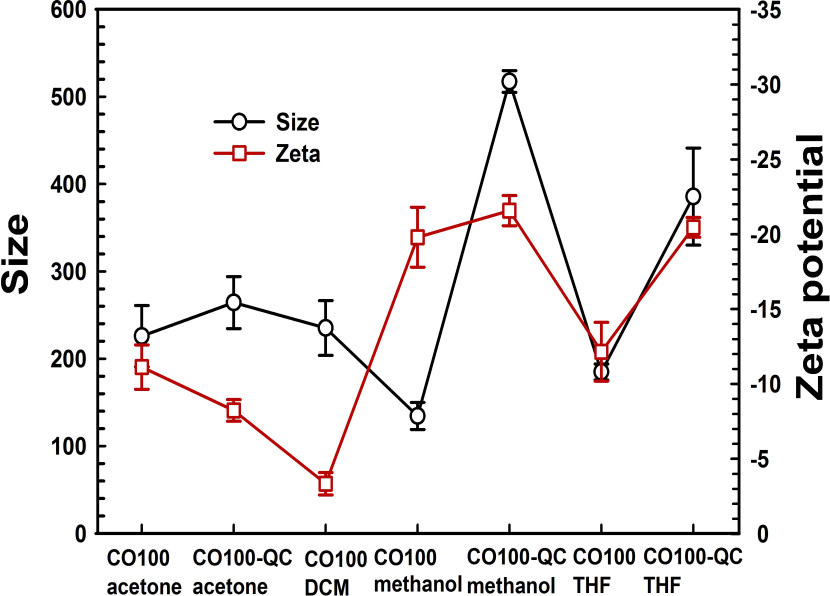


**Figure s8.** Size and zeta potential of CO100 and CO100- QC complexes with CO: QC weight ratio of 5: 1 prepared by dissolving the CO100 and/ or QC in different solvents (acetone, DCM, methanol and THF) after solvents evaporation in PBS buffer (pH= 7.4). The concentration of CO polymers is 0.01 mg/ ml in the final solution.


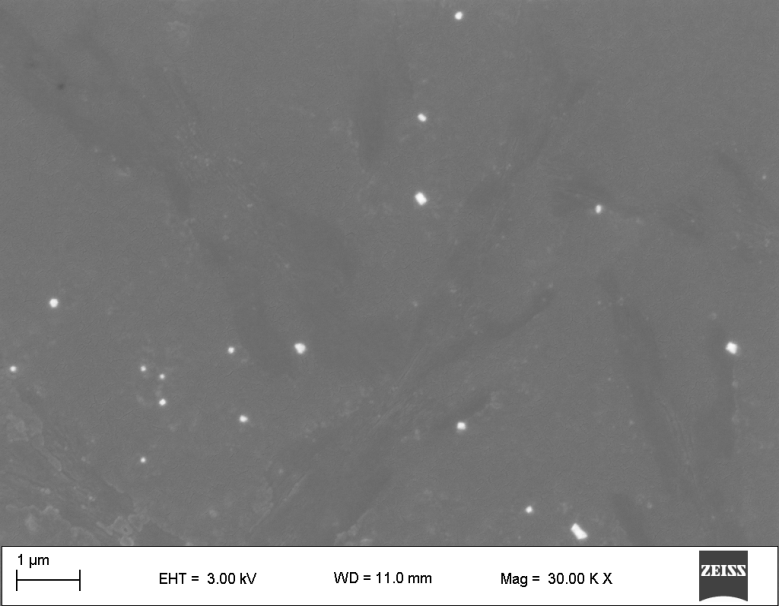


**Figure s9.** SEM image of CO50 polymer micelles prepared by dissolving CO50 in THF after solvent evaporation in DI water to reach 0.01 mg/ ml CO50 in the final solution.


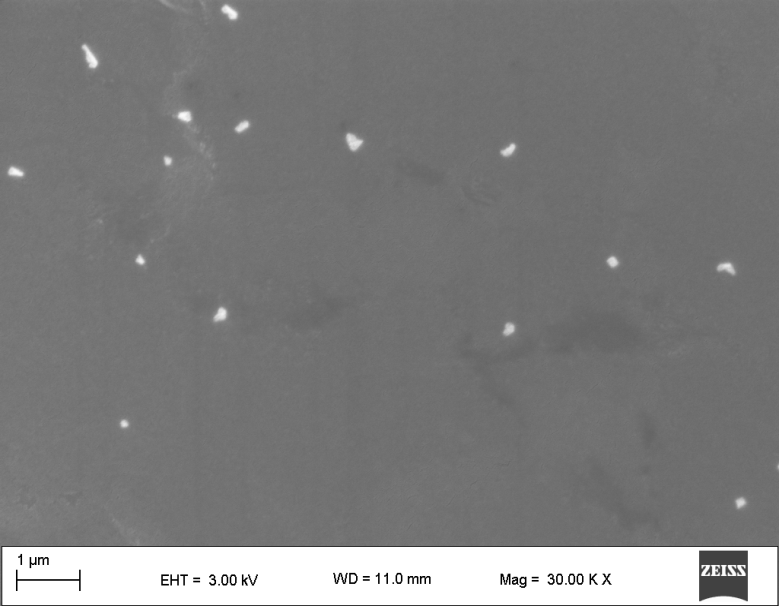


**Figure s10.** SEM image of CO50- QC micelles (CO: QC= 5: 1) prepared by dissolving CO50 and QC in THF after solvent evaporation in DI water to reach 0.01 mg/ ml CO50 in the final solution.


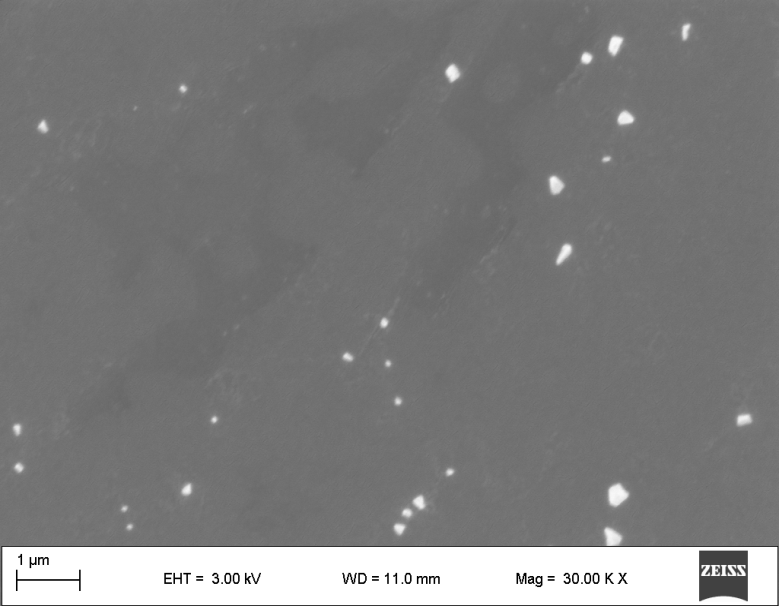


**Figure s11.** SEM image of CO100- QC micelles (CO: QC= 2: 1) prepared by dissolving CO100 and QC in THF after solvent evaporation in DI water to reach 0.01 mg/ ml CO100 in the final solution.


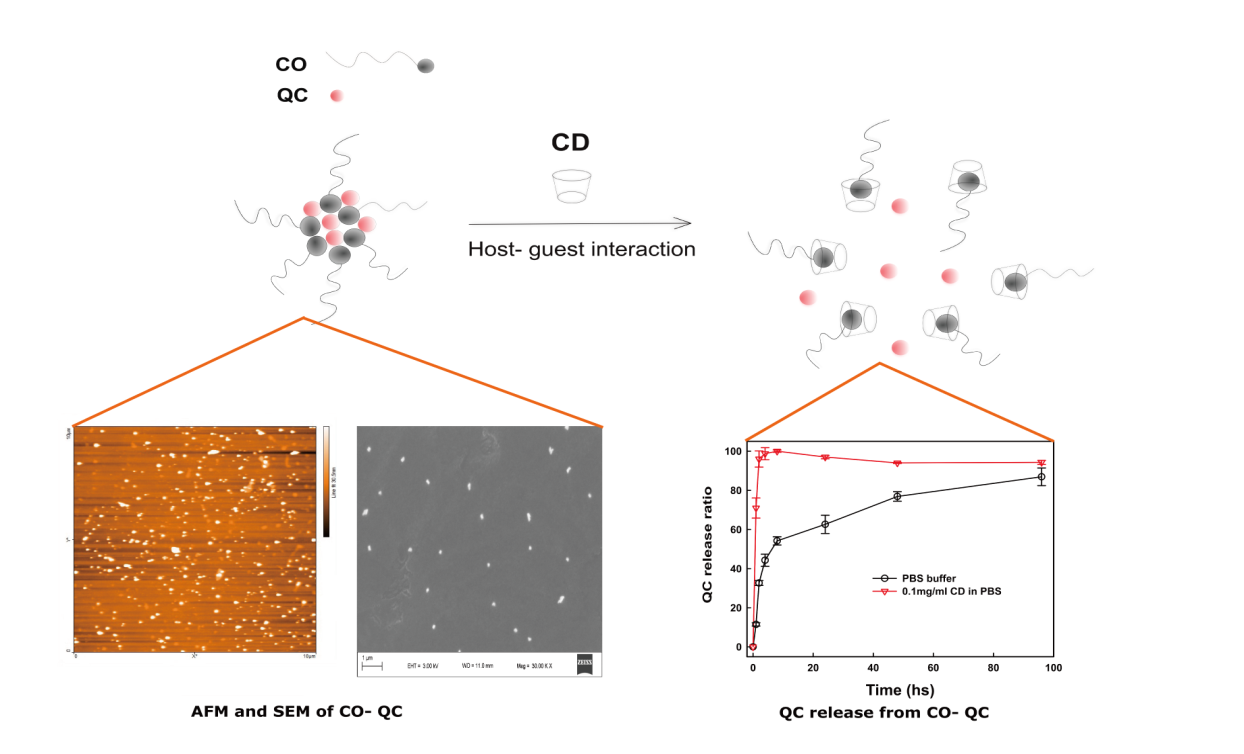


**Table of contents.** Self- assembled and β- cyclodextrin induced drug release of hundreds nanometer sized (AFM and SEM) cholesterol end- capped amphiphilic polymer (CO)- quercetin (QC) micelles.
